# 2-(2-Furylmethyl­ammonio)ethane­sulfonate methanol solvate

**DOI:** 10.1107/S1600536809020005

**Published:** 2009-06-06

**Authors:** Zhong-Xiang Du, Ling-Zhi Wang

**Affiliations:** a Department of Chemistry, Luoyang Normal University, Luoyang, Henan 471022, People’s Republic of China; b Equipment Department, Luoyang Normal University, Luoyang, Henan 471022, People’s Republic of China

## Abstract

The organic mol­ecule of the title compound, C_7_H_11_NO_4_S·CH_3_OH, is a zwitterion and its furan ring displays positional disorder [occupancy 0.563 (5):0.437 (5)]. The crystal structure is extended into a three-dimensional supra­molecular architecture through inter­molecular O—H⋯O and N—H⋯O hydrogen bonds with participation of the methanol solvent mol­ecules.

## Related literature

For a number of reduced or unreduced Schiff base complexes derived from taurine, see: Jiang *et al.* (2004 ▶, 2006 ▶); Li *et al.* (2005 ▶, 2006*a* ▶,*b* ▶, 2007**a* ▶,b* ▶, 2008*a* ▶,*b* ▶); Liao *et al.* (2007 ▶); Zeng *et al.* (2003 ▶); Zhang *et al.* (2005 ▶). For the crystal stucture of a similar compound, 2-(2-pyridylmethyl­ammonio) ethanesulfonate dihydrate, see: Li *et al.* (2006*b* ▶).
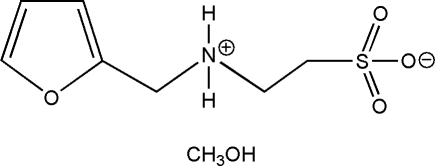


## Experimental

### 

#### Crystal data

C_7_H_11_NO_4_S·CH_4_O
*M* *_r_* = 237.27
Monoclinic, 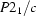

*a* = 10.729 (10) Å
*b* = 9.174 (8) Å
*c* = 11.270 (10) Å
β = 91.964 (10)°
*V* = 1108.6 (17) Å^3^
*Z* = 4
Mo *K*α radiation
μ = 0.29 mm^−1^
*T* = 294 K
0.39 × 0.23 × 0.19 mm

#### Data collection

Bruker APEXII CCD area-detector diffractometer
Absorption correction: multi-scan (*SADABS*; Sheldrick, 1996 ▶) *T*_min_ = 0.894, *T*_max_ = 0.946
7971 measured reflections
2056 independent reflections
1675 reflections with *I* > 2σ(*I*)
*R*_int_ = 0.025

#### Refinement

*R*[*F* ^2^ > 2σ(*F* ^2^)] = 0.041
*wR*(*F* ^2^) = 0.109
*S* = 1.06
2056 reflections
131 parameters
H-atom parameters constrained
Δρ_max_ = 0.34 e Å^−3^
Δρ_min_ = −0.31 e Å^−3^

### 

Data collection: *APEX2* (Bruker, 2004 ▶); cell refinement: *SAINT* (Bruker, 2004 ▶); data reduction: *SAINT*; program(s) used to solve structure: *SHELXS97* (Sheldrick, 2008 ▶); program(s) used to refine structure: *SHELXL97* (Sheldrick, 2008 ▶); molecular graphics: *SHELXTL* (Sheldrick, 2008 ▶); software used to prepare material for publication: *SHELXTL*.

## Supplementary Material

Crystal structure: contains datablocks global, I. DOI: 10.1107/S1600536809020005/at2791sup1.cif

Structure factors: contains datablocks I. DOI: 10.1107/S1600536809020005/at2791Isup2.hkl

Additional supplementary materials:  crystallographic information; 3D view; checkCIF report

## Figures and Tables

**Table 1:** Hydrogen-bond geometry (Å, °)

*D*—H⋯*A*	*D*—H	H⋯*A*	*D*⋯*A*	*D*—H⋯*A*
N1—H1*A*⋯O6^i^	0.90	1.92	2.767 (3)	156
N1—H1*B*⋯O2^ii^	0.90	2.15	2.940 (3)	147
N1—H1*B*⋯O2^iii^	0.90	2.39	3.039 (3)	129
O6—H6⋯O4^iv^	0.82	1.90	2.720 (3)	175

Symmetry codes: (i) 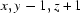; (ii) 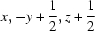; (iii) 
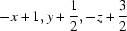
; (iv) 
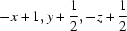
.

**Fig. 1.**
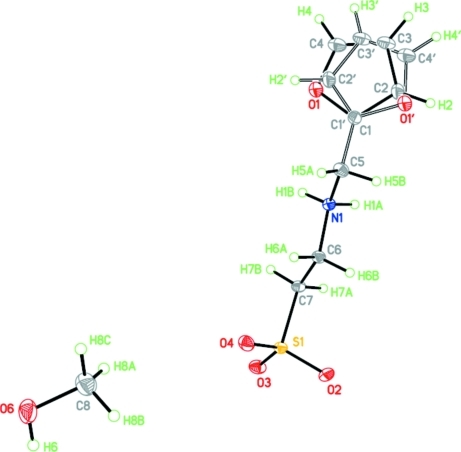
Molecular structure of (I), with displacement ellipsoids drawn at the 15% probability level.

**Fig. 2.**
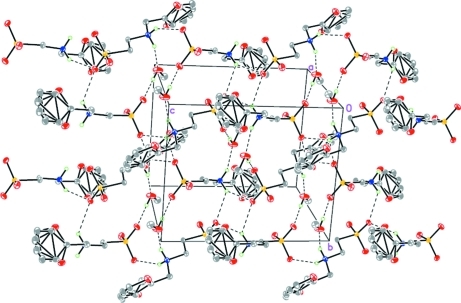
The crystal packing of (I), showing hydrogen bonds as dashed lines in bc plane. H atoms on C atoms have been omitted.

**Fig. 3.**
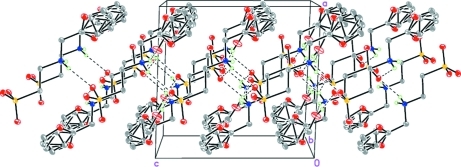
The crystal packing of (I), showing hydrogen bonds as dashed lines in ac plane. H atoms on C atoms have been omitted.

## supplementary crystallographic information

### Comment

In the previous literatures, a number of reduced or unreduced Schiff base complexes derived from taurine have been reported (Jiang *et al.*, 2004, 2006; Li *et al.*, 2005, 2006*a*, 2007a,b, 2008a,b); Liao *et al.*, 2007; Zeng *et al.*, 2003; Zhang *et al.*, 2005), and they have shown novel chain (Li *et al.*, 2007*b*), cubical (Li *et al.*, 2008*a*) and isomeric (Li *et al.*, 2008*b*) structures except for the commonly seen mononuclear or binuclear compounds. Taurine, an amino acid containing sulfur, is indispensable to human beings and has important physiological functions. However, there have been sparse reports on the crystal structures of the corresponding free Schiff base ligands so far. In this paper, we report the crystal structure of a reduced Schiff base from taurine, (I) (Fig. 1).

The H atom of the sulfonic acid group is transferred to the amino N atom, forming the zwitterionic amino acid. This structure is completely similar to that of 2-(2-pyridylmethylammonio)ethanesulfonate dihydrate (Li *et al.*, 2006*b*), where the H atom of the sulfonic acid group is also transferred to the amino N atom. The difference between them is that the furan ring here is positionally disordered. The two positions of furan ring have a dihedral angle of 180°. Other bond length and angles are in good agreement. Methanol molecules are involved in hydrogen bonds both as donors and acceptors, whereas ammonium acts only as a double donor (Table 1, Fig.2). Fig. 3 shows the crystal packing of (I), with hydrogen bonds as dashed lines in *ac* plane. The crystal of (I) is stabilized *via* these intermolecular hydrogen bonding interactions.

### Experimental

Furan-2-carbaldehyde (0.96 g, 10 mmol) in MeOH (10 ml) was dropwise added to a solution of 2-aminoethanesulfonic acid (1.25 g, 10 mmol) in methanol (10 ml) containing KOH (0.56 g, 10 mmol). The yellow solution was stirred for about 2 h at room temperature prior to cooling in an ice bath. The intermediate Schiff base that formed was reduced with an excess of KBH_4_ (0.79 g, 15 mmol). The yellow colour slowly discharged, and after 3 h the solution was adjusted with concentrated HCl to pH = 6.0. The resulting white solid was filtered off, washed with anhydrous methanol and diethyl ether. The obtained solid was dissolved in a ethanol-methanol mixture (1:1 *v*/*v*, 20 ml) and heated. When cooling, colourless granular-shaped crystals were obtained in a yield of 76%. Analysis, found: C 40.42, H 6.37, N 5.85, S 13.55%; C_8_H_15_NO_5_S requires: C 40.50, H 6.33, N 5.91, S 13.50%. IR (KBr,ν, cm^-1^): 768.7[γ(C═C-H)], 741.0(γCH_2_); 1210.1, 1147.5, 1040.8(ν SO_3_^-^); 1607.6(ν C═C); 3428.4(ν O-H); 3098.8, 3021.3(ν N-H).

### Refinement

The H atoms bonded to C and N atoms were positioned geometrically with C—H distance of 0.93–0.97Å and N—H distances of 0.900 Å, and treated as riding atoms, with *U*_iso_(H) = 1.2 or 1.5*U*_eq_(C, N). The O—H hydrogen atom was located in a difference Fourier map and their positional and isotropic displacement parameters were refined; the applied restraint of the O—H distance wasere 0.820 Å, with *U*_iso_(H) = 1.5*U*_eq_(O).

### Crystal data

**Table:**

C_7_H_11_NO_4_S·CH_4_O	*F*(000) = 504
*M**_r_* = 237.27	*D*_x_ = 1.422 Mg m^−^^3^
Monoclinic, *P*2_1_/*c*	Mo *K*α radiation, λ = 0.71073 Å
Hall symbol: -P 2ybc	Cell parameters from 2624 reflections
*a* = 10.729 (10) Å	θ = 2.9–26.3°
*b* = 9.174 (8) Å	µ = 0.29 mm^−^^1^
*c* = 11.27 (1) Å	*T* = 294 K
β = 91.964 (10)°	Granular, colourless
*V* = 1108.6 (17) Å^3^	0.39 × 0.23 × 0.19 mm
*Z* = 4	

### Data collection

**Table:**

Bruker APEXII CCD area-detector diffractometer	2056 independent reflections
Radiation source: fine-focus sealed tube	1675 reflections with *I* > 2σ(*I*)
graphite	*R*_int_ = 0.025
φ and ω scans	θ_max_ = 25.5°, θ_min_ = 2.9°
Absorption correction: multi-scan (*SADABS*; Sheldrick, 1996)	*h* = −12→12
*T*_min_ = 0.894, *T*_max_ = 0.946	*k* = −11→11
7971 measured reflections	*l* = −13→13

### Refinement

**Table:**

Refinement on *F*^2^	Primary atom site location: structure-invariant direct methods
Least-squares matrix: full	Secondary atom site location: difference Fourier map
*R*[*F*^2^ > 2σ(*F*^2^)] = 0.041	Hydrogen site location: inferred from neighbouring sites
*wR*(*F*^2^) = 0.109	H-atom parameters constrained
*S* = 1.06	*w* = 1/[σ^2^(*F*_o_^2^) + (0.0435*P*)^2^ + 0.7985*P*] where *P* = (*F*_o_^2^ + 2*F*_c_^2^)/3
2056 reflections	(Δ/σ)_max_ = 0.001
131 parameters	Δρ_max_ = 0.34 e Å^−^^3^
0 restraints	Δρ_min_ = −0.31 e Å^−^^3^

### Special details

**Table:**

Geometry. All e.s.d.'s (except the e.s.d. in the dihedral angle between two l.s. planes) are estimated using the full covariance matrix. The cell e.s.d.'s are taken into account individually in the estimation of e.s.d.'s in distances, angles and torsion angles; correlations between e.s.d.'s in cell parameters are only used when they are defined by crystal symmetry. An approximate (isotropic) treatment of cell e.s.d.'s is used for estimating e.s.d.'s involving l.s. planes.
Refinement. Refinement of *F*^2^ against ALL reflections. The weighted *R*-factor *wR* and goodness of fit *S* are based on *F*^2^, conventional *R*-factors *R* are based on *F*, with *F* set to zero for negative *F*^2^. The threshold expression of *F*^2^ > σ(*F*^2^) is used only for calculating *R*-factors(gt) *etc*. and is not relevant to the choice of reflections for refinement. *R*-factors based on *F*^2^ are statistically about twice as large as those based on *F*, and *R*- factors based on ALL data will be even larger.

### Fractional atomic coordinates and isotropic or equivalent isotropic displacement parameters (Å^2^)

**Table:**

	*x*	*y*	*z*	*U*_iso_*/*U*_eq_	Occ. (<1)
C1	0.15887 (16)	0.40087 (19)	1.03366 (18)	0.0405 (5)	0.563 (5)
C2	0.06631 (19)	0.3384 (3)	1.0916 (3)	0.0555 (12)	0.563 (5)
H2	0.0190	0.2592	1.0650	0.067*	0.563 (5)
C3	0.0522 (3)	0.4135 (4)	1.2010 (2)	0.0573 (19)	0.563 (5)
H3	−0.0047	0.3950	1.2595	0.069*	0.563 (5)
C4	0.1395 (4)	0.5157 (4)	1.1997 (2)	0.0619 (14)	0.563 (5)
H4	0.1532	0.5825	1.2608	0.074*	0.563 (5)
O1	0.2068 (3)	0.5129 (2)	1.0994 (2)	0.0578 (9)	0.563 (5)
O1'	0.0572 (2)	0.3157 (3)	1.0589 (3)	0.0578 (9)	0.437 (5)
C1'	0.15442 (16)	0.40368 (19)	1.03098 (18)	0.0405 (5)	0.437 (5)
C2'	0.1761 (2)	0.4964 (2)	1.11987 (19)	0.0555 (12)	0.437 (5)
H2'	0.2380	0.5675	1.1222	0.067*	0.437 (5)
C3'	0.0903 (3)	0.4705 (4)	1.2108 (2)	0.0573 (19)	0.437 (5)
H3'	0.0832	0.5187	1.2828	0.069*	0.437 (5)
C4'	0.0230 (3)	0.3605 (4)	1.1674 (3)	0.0619 (14)	0.437 (5)
H4'	−0.0418	0.3180	1.2078	0.074*	0.437 (5)
C5	0.2106 (2)	0.3793 (3)	0.9149 (2)	0.0455 (6)	
H5A	0.1911	0.4519	0.8546	0.055*	
H5B	0.1656	0.2896	0.8977	0.055*	
C6	0.3852 (2)	0.2834 (3)	0.80019 (19)	0.0389 (5)	
H6A	0.3740	0.3697	0.7512	0.047*	
H6B	0.3372	0.2049	0.7635	0.047*	
C7	0.5215 (2)	0.2421 (3)	0.80631 (19)	0.0385 (5)	
H7A	0.5316	0.1509	0.8491	0.046*	
H7B	0.5684	0.3165	0.8497	0.046*	
N1	0.33791 (17)	0.3130 (2)	0.92132 (16)	0.0366 (4)	
H1A	0.3355	0.2290	0.9624	0.044*	
H1B	0.3908	0.3739	0.9604	0.044*	
O2	0.51984 (16)	0.09645 (19)	0.60803 (14)	0.0475 (4)	
O3	0.71519 (15)	0.2028 (2)	0.67984 (16)	0.0547 (5)	
O4	0.54838 (17)	0.35667 (19)	0.59804 (16)	0.0541 (5)	
S1	0.58193 (5)	0.22288 (6)	0.66156 (5)	0.03640 (19)	
C8	0.1847 (3)	0.9586 (4)	0.0474 (3)	0.0661 (8)	
H8A	0.2158	0.9121	0.1187	0.099*	
H8B	0.1539	0.8861	−0.0075	0.099*	
H8C	0.1184	1.0241	0.0660	0.099*	
O6	0.2791 (2)	1.0353 (2)	−0.0028 (3)	0.0841 (8)	
H6	0.3274	0.9783	−0.0334	0.126*	

### Atomic displacement parameters (Å^2^)

**Table:**

	*U*^11^	*U*^22^	*U*^33^	*U*^12^	*U*^13^	*U*^23^
C1	0.0334 (12)	0.0395 (13)	0.0489 (14)	0.0009 (10)	0.0043 (10)	−0.0021 (11)
C2	0.054 (3)	0.057 (3)	0.056 (3)	−0.021 (2)	0.008 (2)	−0.008 (2)
C3	0.053 (3)	0.075 (5)	0.0450 (19)	0.007 (3)	0.015 (2)	−0.008 (3)
C4	0.065 (3)	0.071 (3)	0.051 (2)	0.007 (3)	0.016 (2)	−0.007 (2)
O1	0.0508 (17)	0.0572 (19)	0.0662 (18)	−0.0150 (15)	0.0137 (14)	−0.0136 (15)
O1'	0.0508 (17)	0.0572 (19)	0.0662 (18)	−0.0150 (15)	0.0137 (14)	−0.0136 (15)
C1'	0.0334 (12)	0.0395 (13)	0.0489 (14)	0.0009 (10)	0.0043 (10)	−0.0021 (11)
C2'	0.054 (3)	0.057 (3)	0.056 (3)	−0.021 (2)	0.008 (2)	−0.008 (2)
C3'	0.053 (3)	0.075 (5)	0.0450 (19)	0.007 (3)	0.015 (2)	−0.008 (3)
C4'	0.065 (3)	0.071 (3)	0.051 (2)	0.007 (3)	0.016 (2)	−0.007 (2)
C5	0.0374 (13)	0.0510 (15)	0.0482 (14)	0.0053 (11)	0.0042 (10)	0.0031 (11)
C6	0.0411 (12)	0.0455 (14)	0.0302 (11)	−0.0025 (10)	0.0026 (9)	−0.0033 (10)
C7	0.0417 (12)	0.0456 (13)	0.0282 (11)	0.0026 (10)	0.0018 (9)	−0.0035 (10)
N1	0.0373 (10)	0.0374 (10)	0.0351 (10)	−0.0016 (8)	0.0036 (8)	−0.0042 (8)
O2	0.0572 (11)	0.0450 (10)	0.0400 (9)	−0.0009 (8)	−0.0020 (8)	−0.0118 (7)
O3	0.0383 (9)	0.0731 (13)	0.0530 (11)	0.0057 (9)	0.0040 (8)	−0.0093 (9)
O4	0.0659 (12)	0.0465 (11)	0.0507 (10)	0.0079 (9)	0.0144 (9)	0.0144 (8)
S1	0.0405 (3)	0.0390 (3)	0.0299 (3)	0.0022 (2)	0.0039 (2)	−0.0017 (2)
C8	0.0571 (17)	0.068 (2)	0.074 (2)	−0.0017 (15)	0.0079 (15)	0.0062 (16)
O6	0.0751 (15)	0.0432 (11)	0.137 (2)	0.0023 (11)	0.0486 (14)	0.0045 (13)

### Geometric parameters (Å, °)

**Table:**

C1—C2	1.3365 (17)	C5—H5A	0.9700
C1—O1	1.3579 (17)	C5—H5B	0.9700
C1—C5	1.479 (3)	C6—N1	1.497 (3)
C2—C3	1.4241 (19)	C6—C7	1.510 (3)
C2—H2	0.9300	C6—H6A	0.9700
C3—C4	1.3255 (17)	C6—H6B	0.9700
C3—H3	0.9300	C7—S1	1.785 (3)
C4—O1	1.3616 (18)	C7—H7A	0.9700
C4—H4	0.9300	C7—H7B	0.9700
O1'—C4'	1.3528 (18)	N1—H1A	0.9000
O1'—C1'	1.3636 (18)	N1—H1B	0.9000
C1'—C2'	1.3288 (17)	O2—S1	1.4579 (19)
C1'—C5	1.476 (3)	O3—S1	1.449 (2)
C2'—C3'	1.4210 (18)	O4—S1	1.460 (2)
C2'—H2'	0.9300	C8—O6	1.371 (3)
C3'—C4'	1.3242 (17)	C8—H8A	0.9600
C3'—H3'	0.9300	C8—H8B	0.9600
C4'—H4'	0.9300	C8—H8C	0.9600
C5—N1	1.494 (3)	O6—H6	0.8200
			
C2—C1—O1	109.4	N1—C5—H5B	96.4
C2—C1—C5	133.87 (18)	H5A—C5—H5B	110.4
O1—C1—C5	116.63 (19)	N1—C6—C7	111.21 (18)
C1—C2—C3	108.6	N1—C6—H6A	109.4
C1—C2—H2	125.7	C7—C6—H6A	109.4
C3—C2—H2	125.7	N1—C6—H6B	109.4
C4—C3—C2	103.7	C7—C6—H6B	109.4
C4—C3—H3	128.2	H6A—C6—H6B	108.0
C2—C3—H3	128.2	C6—C7—S1	111.39 (15)
C3—C4—O1	113.0	C6—C7—H7A	109.3
C3—C4—H4	123.5	S1—C7—H7A	109.3
O1—C4—H4	123.5	C6—C7—H7B	109.3
C1—O1—C4	105.4	S1—C7—H7B	109.3
C4'—O1'—C1'	105.2	H7A—C7—H7B	108.0
C2'—C1'—O1'	108.7	C5—N1—C6	111.57 (17)
C2'—C1'—C5	134.33 (19)	C5—N1—H1A	109.3
O1'—C1'—C5	116.98 (18)	C6—N1—H1A	109.3
C1'—C2'—C3'	109.6	C5—N1—H1B	109.3
C1'—C2'—H2'	125.2	C6—N1—H1B	109.3
C3'—C2'—H2'	125.2	H1A—N1—H1B	108.0
C4'—C3'—C2'	102.7	O3—S1—O2	113.08 (11)
C4'—C3'—H3'	128.6	O3—S1—O4	113.72 (12)
C2'—C3'—H3'	128.6	O2—S1—O4	111.38 (12)
C3'—C4'—O1'	113.8	O3—S1—C7	105.68 (11)
C3'—C4'—H4'	123.1	O2—S1—C7	106.36 (11)
O1'—C4'—H4'	123.1	O4—S1—C7	105.89 (11)
C1'—C5—N1	114.81 (19)	O6—C8—H8A	109.5
C1—C5—N1	112.44 (19)	O6—C8—H8B	109.5
C1'—C5—H5A	115.6	H8A—C8—H8B	109.5
C1—C5—H5A	117.6	O6—C8—H8C	109.5
N1—C5—H5A	119.2	H8A—C8—H8C	109.5
C1'—C5—H5B	95.2	H8B—C8—H8C	109.5
C1—C5—H5B	95.5	C8—O6—H6	109.5
			
O1—C1—C2—C3	0.2	O1'—C1'—C5—C1	−105.89 (12)
C5—C1—C2—C3	−175.1 (3)	C2'—C1'—C5—N1	71.6 (3)
C1—C2—C3—C4	−0.4	O1'—C1'—C5—N1	−108.9 (2)
C2—C3—C4—O1	0.4	C2—C1—C5—C1'	72.75 (19)
C2—C1—O1—C4	0.0	O1—C1—C5—C1'	−102.27 (13)
C5—C1—O1—C4	176.2 (2)	C2—C1—C5—N1	−110.2 (2)
C3—C4—O1—C1	−0.3	O1—C1—C5—N1	74.7 (2)
C4'—O1'—C1'—C2'	0.0	N1—C6—C7—S1	−174.72 (15)
C4'—O1'—C1'—C5	−179.6 (2)	C1'—C5—N1—C6	176.54 (19)
O1'—C1'—C2'—C3'	−0.2	C1—C5—N1—C6	176.41 (18)
C5—C1'—C2'—C3'	179.3 (3)	C7—C6—N1—C5	169.8 (2)
C1'—C2'—C3'—C4'	0.3	C6—C7—S1—O3	172.61 (17)
C2'—C3'—C4'—O1'	−0.3	C6—C7—S1—O2	−66.9 (2)
C1'—O1'—C4'—C3'	0.2	C6—C7—S1—O4	51.7 (2)
C2'—C1'—C5—C1	74.6 (2)		

### Hydrogen-bond geometry (Å, °)

**Table:**

*D*—H···*A*	*D*—H	H···*A*	*D*···*A*	*D*—H···*A*
N1—H1A···O6^i^	0.90	1.92	2.767 (3)	156
N1—H1B···O2^ii^	0.90	2.15	2.940 (3)	147
N1—H1B···O2^iii^	0.90	2.39	3.039 (3)	129
O6—H6···O4^iv^	0.82	1.90	2.720 (3)	175

Symmetry codes: (i) *x*, *y*−1, *z*+1; (ii) *x*, −*y*+1/2, *z*+1/2; (iii) −*x*+1, *y*+1/2, −*z*+3/2; (iv) −*x*+1, *y*+1/2, −*z*+1/2.
